# Highly Sensitive Bacteria Quantification Using Immunomagnetic Separation and Electrochemical Detection of Guanine-Labeled Secondary Beads

**DOI:** 10.3390/s150512034

**Published:** 2015-05-22

**Authors:** Harikrishnan Jayamohan, Bruce K. Gale, Bj Minson, Christopher J. Lambert, Neil Gordon, Himanshu J. Sant

**Affiliations:** 1 Department of Mechanical Engineering, University of Utah, Salt Lake City, UT 84112, USA; E-Mail: bruce.gale@utah.edu; 2 Espira Inc., 825 N 300 W Suite N-223, Salt Lake City, UT 84103, USA; E-Mail: bjminson@gmail.com; 3 Department of Bioengineering, University of Utah, Salt Lake City, UT 84112, USA; E-Mail: chris.lambert@utah.edu; 4 Guanine Inc., Salt Lake City, UT 84103, USA; E-Mail: neil.gordon@guanineinc.com

**Keywords:** *Escherichia coli* O157:H7 detection, biosensors, pathogen detection, electrochemical detection, differential pulse voltammetry, immunomagnetic separation

## Abstract

In this paper, we report the ultra-sensitive indirect electrochemical detection of *E. coli* O157:H7 using antibody functionalized primary (magnetic) beads for capture and polyguanine (polyG) oligonucleotide functionalized secondary (polystyrene) beads as an electrochemical tag. Vacuum filtration in combination with *E. coli* O157:H7 specific antibody modified magnetic beads were used for extraction of *E. coli* O157:H7 from 100 mL samples. The magnetic bead conjugated *E. coli* O157:H7 cells were then attached to polyG functionalized secondary beads to form a sandwich complex (magnetic bead/*E. coli*/ secondary bead). While the use of magnetic beads for immuno-based capture is well characterized, the use of oligonucleotide functionalized secondary beads helps combine amplification and potential multiplexing into the system. The antibody functionalized secondary beads can be easily modified with a different antibody to detect other pathogens from the same sample and enable potential multiplexing. The polyGs on the secondary beads enable signal amplification up to 10^8^ guanine tags per secondary bead (7.5 × 10^6^ biotin-FITC per secondary bead, 20 guanines per oligonucleotide) bound to the target (*E. coli*). A single-stranded DNA probe functionalized reduced graphene oxide modified glassy carbon electrode was used to bind the polyGs on the secondary beads. Fluorescent imaging was performed to confirm the hybridization of the complex to the electrode surface. Differential pulse voltammetry (DPV) was used to quantify the amount of polyG involved in the hybridization event with tris(2,2′-bipyridine)ruthenium(II) ( ${\text{Ru}(\text{bpy})}_{3}^{2+}$) as the mediator. The amount of polyG signal can be correlated to the amount of *E. coli* O157:H7 in the sample. The method was able to detect concentrations of *E. coli* O157:H7 down to 3 CFU/100 mL, which is 67 times lower than the most sensitive technique reported in literature. The signal to noise ratio for this work was 3. We also demonstrate the use of the protocol for detection of *E. coli* O157:H7 seeded in waste water effluent samples.

## 1. Introduction

Food and water-borne diseases are a major source of concern worldwide. According to the World Health Organization, gastrointestinal infections kill around 2.2 million people globally each year [1]. The pathogenic strains of *E. coli* such as O157:H7 are a major source of food and water-borne disease outbreaks around the world [2]. As Escherichia coli (*E. coli*) is a bacterium found in the lower intestine of warm-blooded organisms, it is considered an indicator organism to test environmental samples for fecal contamination [3]. Even low levels of *E. coli* O157:H7 (10–100 viable organisms) can cause human infections [2,4].

Current methods of *E. coli* detection involve conventional techniques like membrane filtration, plate counting [5], turbidimetry and multiple-tube fermentation. These techniques though reliable, are time consuming (24–48 h), complex and require trained personnel [6]. Additionally, none of these techniques are suitable for point-of-use, which is essential in monitoring pathogenic bacteria in geographically remote locations. Recently, biosensing methods including electronic [7], mass-based [8], optical [9,10] and electrochemical (EC) techniques [11–13] have been applied for detecting pathogenic bacteria [4,14]. Among these, EC methods are increasingly relied upon due to advantages like simplicity, accuracy, fast response, low cost, and portability [4,6]. EC sensors can also be integrated on a chip and can be multiplexed for detecting multiple pathogens and strains [15].

**Figure 1:**
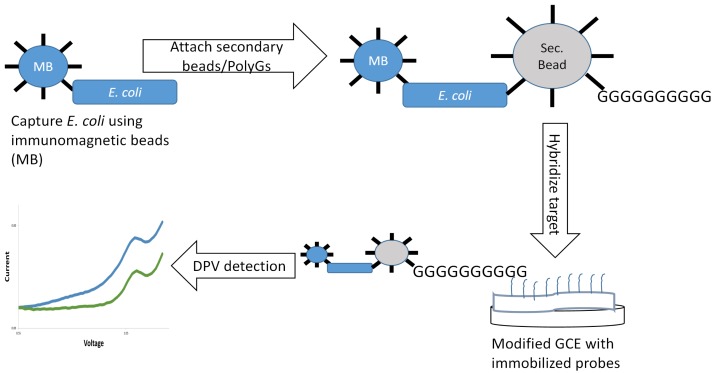
Working principle of the *E. coli* detection mechanism.

EC detection has been shown to be very sensitive in the detection of *E. coli*. Han *et al*. reported an EC immunosensor for *E. coli* using graphene oxide-Ag nanoparticle composite labels with limits of detection down to 10 colony-forming units (CFU) per mL [6]. dos Santos recently reported a limit of detection of 2 CFU/mL using an electrochemical impedance spectroscopy based immunosensor [4]. Note, though that, environmental standards for *E. coli* in water are mostly defined for 100 mL samples. For instance, the U.S Environmental Protection Agency defines protocols for testing *E. coli* limits in the Clean Water Act for 100 mL sampling volumes [16–18], most likely because 1 mL would not be statistically representative of the volumes involved. In addition, real world samples experience interference from the sample matrix and background microflora, making isolation and detection of bacterial pathogens more challenging [19]. We have coupled immunomagnetic capture and EC detection to enable sensitive detection of *E. coli* from waste water effluent (Figure 1).

Immunoaffinity capture techniques, like immunomagnetic separations (IMS), have been applied to isolate and concentrate *E. coli* from water samples [20]. These techniques purify *E. coli* and remove contaminants that might interfere with *E. coli* detection signals during subsequent detection assays [19]. Sample purification also reduces the incidence of false positive and false negative test results by removing virtually all inhibiting materials that could be incorrectly detected. IMS has also been incorporated into microfluidic point-of-use systems and the process can be automated [21,22]. Zhu *et al*. applied IMS coupled with fluorescent detection (using a spectrofluorometer) of *E. coli* O157:H7 and obtained a limit of detection of 10 CFU/mL [19]. However fluorescent detection requires related optical detection equipment, which is often not miniaturized making the approach less amenable for point-of-use [23–25]. Immunomagnetic beads have been used to capture *E. coli* and subsequently detect the bacteria using electrochemical methods without secondary bead based amplification [26]. To achieve ultra-sensitive detection of pathogens, a signal amplification step was incorporated to the IMS. Nam *et al*. reported the use of immunomagnetic capture combined with secondary beads (bio-barcodes) for signal amplification in the detection of DNA and proteins [27,28]. The work relied on optical methods for detection of the bio-barcodes.

**Figure 2:**
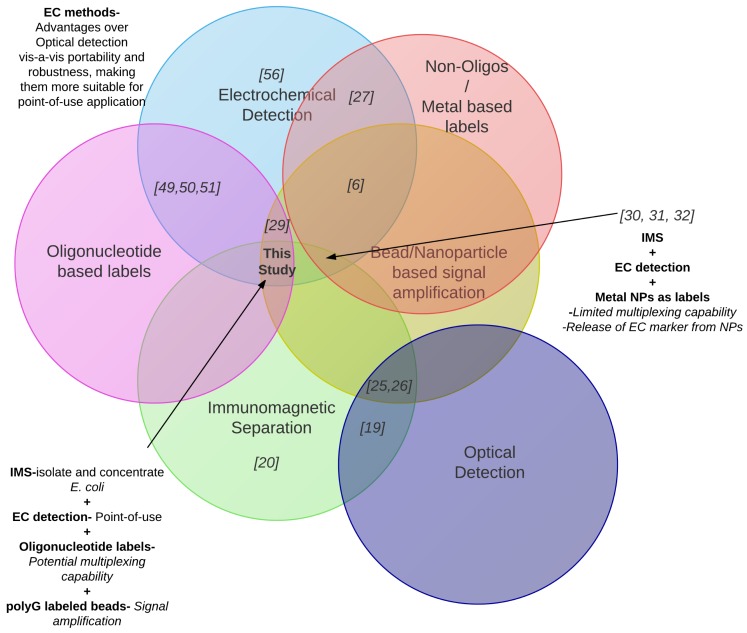
Review of recent point-of-use methods used for detection of proteins and DNA sequences.

The use of electrochemical methods using bio-barcodes has been reported for the detection of proteins and DNA, including DNA from pathogens (Figure 2) [29–34]. Some of these methods have relied on non-oligonucleotide based electrochemical labels for detection. For instance, Ding *et al*. reported the use of cadmium sulfide nanoparticles as electrochemical labels for the detection of human *α*-fetoprotein [32]. Zhang *et al*. applied lead sulfide and cadmium sulfide as electrochemical labels for the detection of *Bacillus anthracis and Salmonella enteritidis* [33]. The use of metal nanoparticles as electrochemical labels has disadvantages vis-a-vis oligonucleotides with regard to multiplexing capabilities. The number of entities that can be simultaneously detected is restricted by the number of metals that have a peak potential (ΔE*_p_*) within a given electrochemical range. For instance, the use of Pb^2+^ (anodic oxidation ΔE*_p_* = −0.61 V) and Cd^2+^ (anodic oxidation ΔE*_p_* = −0.87 V) as electrochemical labels restricts the use of any other label with peak potential in between these due to issues with peak separation. In contrast, using oligonucleotide electrochemical labels provides multiplexing possibilities limited only by the number of electrodes with complimentary probes on them. The use of metal nanoparticles also involves an additional step of dissolution of the EC marker from the beads onto the electrodes for detection. Wang *et al*. reported the use of guanine tagged polymeric beads for the detection of proteins [30]. However the guanine tags had to be released from the beads for detection using potentiometric stripping. By releasing the guanine, there was no possibility of distinguishing the tags from different analytes for potential multiplexing. This method, although it enables amplification of the detection signal, does not enable multiplexing. In contrast, keeping oligonucleotide EC labels intact provides multiplexing possibilities with complementary probes on individual working electrodes assigned to specific analytes.

In this paper, we report the use of immunomagnetic capture coupled with amplification and indirect EC detection of *E. coli* O157:H7 on an electrochemically reduced graphene oxide glassy carbon electrode (RGO-GCE). *E. coli* O157:H7 specific antibodies coated magnetic beads were used to capture *E. coli* O157:H7 strains from water samples. The use of polyG functionalized secondary beads in addition to the magnetic beads incorporates signal amplification and potential multiplexing capability. To enable multiplexing and amplification we use synthetic polyguanine oligonucleotides (polyG) as an EC tag and amplification system. The use of biobarcode based signal amplification enables higher sensitivity due to the large number of DNA strands in each single molecular binding event [27,28,35,36]. The bacteria collected using magnetic beads is attached to another set of *E. coli* O157:H7 antibody functionalized nonmagnetic polystyrene (secondary) beads. These secondary beads have an EC tag (polyGs) that can be correlated to the *E. coli* O157:H7 concentration in the sample. The nonmagnetic secondary beads can be easily modified with a different antibody to capture a different pathogen. By using a different polyG sequence on the secondary beads (and using corresponding complementary probe sequence on the RGO-GCE electrode), the system can be modified to detect multiple pathogens. After washing steps, we transfer this complex (magnetic beads, bacteria, and nonmagnetic beads) to the RGO-GCE electrode. These polyGs are hybridized with complementary probes on the electrode surface and upon an EC scan generate a guanine oxidation signal that is correlated to *E. coli* O157:H7 concentration in the sample. Using the protocol we demonstrate detection of *E. coli* O157:H7 in phosphate buffered solution (PBS) and waste water samples. To the best of our knowledge, this is the first instance of combining IMS with oligonucleotide functionalized secondary bead based amplification for electrochemical detection of pathogens. The reported protocol is highly sensitive and selective, and can be potentially multiplexed for detecting multiple pathogens. The protocol has also been applied in the detection of *E. coli* O157:H7 in waste water samples.

## 2. Experimental Section

### 2.1. Working Principle of the E. coli O157:H7 Sensor

**Figure 3:**
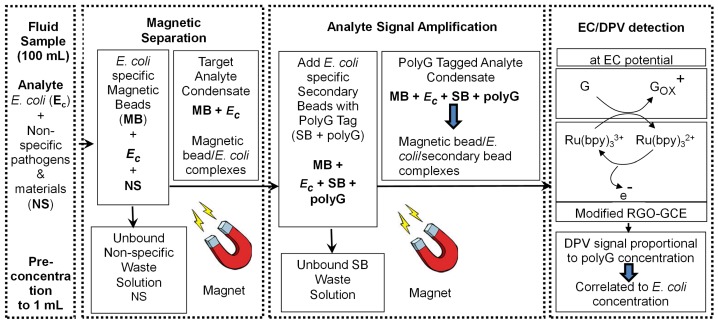
Mechanism of indirect sensing of *E. coli* O157:H7 using IMS and subsequent signal amplification using polyG functionalized secondary beads.

The mechanism of indirect sensing of *E. coli* O157:H7 is illustrated in Figure 3 [37]. The mechanism consists of four steps which are:

(I) Vacuum filtration to pre-concentrate the *E. coli* O157:H7 in 100 mL samples into a 1 mL sample volume
(II) IMS to selectively capture *E. coli* O157:H7
(III) Analyte amplification consisting of an EC polyG tag attached to secondary beads
(IV) EC detection of the polyG tags

In the IMS step, the bacteria sample is concentrated from water sample (PBS or waste water) by filtration and isolated using *E. coli* O157:H7 specific antibody coated magnetic beads. To enable amplification synthetic polyG oligos are used as an EC tag and amplification system. The bacteria collected using magnetic beads is attached to another set of secondary beads containing EC tag (polyG oligos) and can be correlated to the *E. coli* O157:H7 concentration in the sample. The sample is then washed to remove any unbound secondary beads. The magnetic bead/*E. coli*/secondary bead complexes are transferred to the EC detector and the polyGs on the secondary beads are hybridized with complementary probes on the electrode surface. A DPV scan generates a signal corresponding to the polyGs on the secondary beads that is indirectly correlated to *E. coli* O157:H7 concentration in the sample. The probes on the electrode surface are specific to the polyGs on the secondary beads to ensure selectivity.

### 2.2. Apparatus and Reagents

EC deposition and differential pulse voltammetry (DPV) were carried out using a Gamry Reference 600 potentiostat (Gamry Instruments, Warminster, PA, USA). A conventional three-electrode system, which consisted of a modified glassy carbon electrode (GCE-3.0 mm diameter, Catalog no. MF-2012, BASi, West Lafayette, IN, USA) as a working electrode, an Ag/AgCl electrode as a reference electrode and a platinum mesh as an counter electrode, was employed for the DPV and EC deposition.

Graphene oxide for EC deposition was purchased from Graphene Supermarket (Calverton, NY, USA). *E. coli* O157:H7 nonpathogenic strain (Catalog no. 700728) was obtained from ATCC (Manassas, VA, USA). The *E. coli* O157:H7 antibody coated magnetic beads for pathogen extraction were obtained from Invitrogen (Dynabeads MAX *E. coli* O157 kit, Invitrogen, Carlsbad, CA, USA). The streptavidin coated polystyrene (secondary) beads were purchased from Bangs Laboratories (9.78 μm mean diameter, Catalog no. CP01N-11339, Bangs Laboratories Inc., Fishers, IN, USA). Biotin-labeled BacTrace anti-*E. coli* O157:H7 antibody was purchased from Kirkegaard and Perry Laboratories (Catalog no. 16-95-90, KPL Inc., Gaithersburg, MD, USA). Sulfo-NHS (N-hydroxysulfo-succinimide) and EDC (1-ethyl-3(3-dimethly aminopropyl) carbodiimide hydrochloride) were obtained from Pierce/Thermo Fisher Scientific (Rockford, IL, USA). Sodium hydroxide was ordered from Macron Fine Chemicals (Center Valley, PA, USA). Tris(2,2′-bipyridyl)ruthenium(II) chloride hexahydrate (Ru(bpy)_3_Cl_2_) was purchased from Sigma-Aldrich (Catalog no. 224758-1G, St. Louis, MO, USA). The oligonucleotides were obtained from DNA/Peptide synthesis core facility, University of Utah (Salt Lake City, UT, USA).

All reagents were of analytical grade and were used as received without further purification. Ultra-pure deionized (DI) water prepared by Purelab System (ELGA Purelab, UK) was used throughout the experiment.

### 2.3. Culturing of E. coli O157:H7

*E. coli* O157:H7 nonpathogenic strain (Catalog no. 700728) was obtained from ATCC. Using manufacturer-supplied protocols [38], the freeze dried pellet was reconstituted using Difco Nutrient Broth (Catalog no. 234000, Becton Dickinson, Sparks, MD, USA). The pellet was hydrated using 1 mL of the Difco Broth and then placed in 5 mL of additional broth. Then 200 μL was taken from the broth and placed on an agar plate prepared using Difco Nutrient Agar (Catalog no. 213000, Becton Dickinson, Sparks, MD, USA). The broth and agar plate were incubated at 37 °C for 36 h. After the incubation period the broth culture was preserved using a protocol supplied by ATCC. The culture broth was centrifuged at 1000 g for 10 min in order to compact the bacteria into a pellet. The broth supernatant was poured off and 3 mL of broth was added to the pellet. Then, 3 mL of sterilized 20% glycerol (vol/vol) was added to the culture. The culture was then placed in Nalgene Cryogenic vials (Thermo Scientific) and placed at −135 °C for storage. To prepare the samples, the stored *E. coli* O157:H7 was initially plated on agar plates for 16 h and subsequently collected using a sterile pipette tip. The *E. coli* O157:H7 was then vortexed with 10 mL of 1 × PBS solution. About 2 mL of this solution was tested using a spectrophotometer (Biochrom WPA Biowave DNA spectrophotometer) and diluted as necessary to achieve an OD_600_ of 0.1 (corresponding to a concentration of approximately 50 million *E. coli* O157:H7 per mL). The spectrophotometer was calibrated for *E. coli* O157:H7 using a manual cytometer for bacterial counts before use. Then 100 μL of this solution was serially diluted in 1 × PBS buffer to achieve different concentrations of 100 mL samples. The final concentration of *E. coli* O157:H7 was confirmed using plate counting.

### 2.4. Pre-Concentration of E. coli O157:H7 from Seeded PBS Buffer Sample

Vacuum filtration was employed to pre-concentrate the *E. coli* O157:H7 in 100 mL PBS samples into a 1 mL sample volume. A 0.1 μm Durapore membrane filter (Catalog no. VVLP04700, Millipore, Billerica, MA, USA) was securely held in a custom filtration device and attached to a 2000 mL filtering flask (Catalog no. 5340, Pyrex, Corning Inc., Corning, NY, USA). The flask was vacuum pressurized to −55 kPa and the 100 mL of the *E. coli* O157:H7 sample was loaded into a reservoir above the filtration device. The liquid sample was pulled through the filter trapping bacteria and solids larger than 0.1 μm. The filter was then removed from the device, inserted into a 1.5 mL Eppendorf tube containing 1 mL of 1 × PBS and vortexed for a minute to free the bound bacteria. The filter was subsequently removed from the tube and IMS was followed on the 1 mL *E. coli*-PBS buffer sample. The initial and post-filtration *E. coli* O157:H7 samples were plated, incubated at 37 °C for 12 h, and subsequently counted to determine the efficiency of *E. coli* O157:H7 capture during the process.

### 2.5. Immunomagnetic Separation of E. coli O157:H7

*E. coli* O157:H7 specific antibody coated magnetic beads (Dynabeads) were used to extract the *E. coli* O157:H7 from the 1 mL samples [39]. Twenty (μL of magnetic beads (Dynabeads) was added to the tubes containing 1 mL *E. coli* O157:H7 sample, placed on a Mini-Lab Roller (Labnet International Inc., Edison, NJ, USA) rotating mixer, and rotated at 24 rpm for 10 min. The tubes were inserted into a custom built magnetic capture unit for 3 min with occasional inversion to concentrate the beads into a pellet. Hundred μL of the supernatant solution was pipetted onto another agar plate to test for any *E. coli* O157:H7 not captured by the beads. The remainder of the supernatant was carefully pipetted out so as to not disturb the magnetic pellet. The tube was removed from the magnetic capture unit and 1 mL of 1 × Dynabeads wash buffer was added to the tube and returned to the rotating mixer for 3 min. This process of mixing, plating 100 μL, removing supernatant, and washing with 1 mL of 1 × buffer was repeated two more times for a total of 3 wash cycles. After the final wash was removed, 100 μL of 1 × Dynabeads wash buffer was added to the magnetic beads, resuspended and plated on a final agar plate. The plates were incubated at 37 °C for 12 h before being counted to test the efficiency of magnetic bead extraction process.

While calibration of the spectrophotometer with the *E. coli* O157:H7 allowed for relatively accurate predictions of bacteria concentrations in the dilution series, bacteria samples from the dilution series were also plated in order to obtain the most accurate prediction of the original bacteria concentration of the tested sample.

### 2.6. Specificity of Immunomagnetic Separation

Three runs of immunomagnetic separation using *E. coli* O157:H7 specific antibody coated magnetic beads (Dynabeads) were performed on samples of 3000 CFUs of *Salmonella* in 1 mL of 1 × PBS, similar to the protocol mentioned above. The magnetic beads were resuspended and plated on agar plates to determine the amount of *Salmonella* non-specifically bound to the *E. coli* O157:H7 specific antibody coated magnetic beads.

### 2.7. Secondary Beads Functionalization Chemistry

2.5 μL of 50 μM 20 m biotinylated polyG (GGGGGGGGGGGGGGGGGGGG/3′-Biotin) was added to 20 μL of streptavidin coated polystyrene (secondary) beads. Subsequently, 12.5 μL of 1 mg/mL anti-*E*. *coli* O157:H7 antibodies was added to the 20 μL of the polyG functionalized secondary beads [40].

### 2.8. Attachment of Secondary Beads to Magnetic Bead-E. coli O157:H7 Complexes

The magnetic bead and bacteria complex was bound to the *E. coli* O157:H7 antibody functionalized secondary nonmagnetic polystyrene bead. Magnetic bead/*E. coli* O157:H7 complex was resuspended in 20 μL of 1 × PBS and then added to a 20 μL solution of resuspended secondary bead/polyG/antibody complex. The solution was pipet mixed every 5 to 7 min over a 20 min period.

### 2.9. Preparation of the Electrode-Electrodeposition of Graphene Oxide

**Figure 4:**
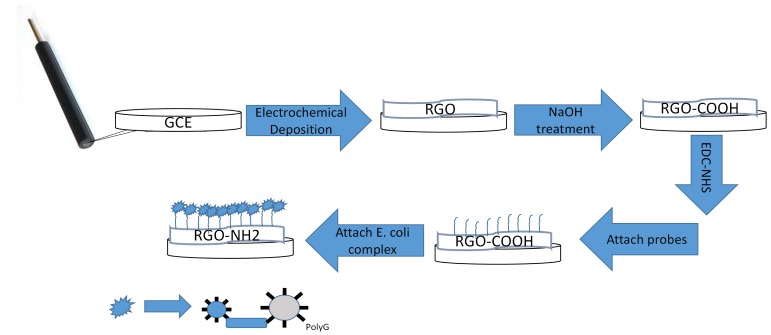
Schematic of GCE preparation for capture of the magnetic bead/*E. coli*/secondary bead complexes.

We have applied electrodeposition to deposit graphene oxide on the bare GCE (Figure 4). Twenty five mg of graphene oxide was added to 50 mL of 0.1 M PBS. The graphene oxide (GO) in solution was exfoliated by ultra-sonication for 30 min to form a homogeneous brown colloidal dispersion with a concentration of 0.5 mg/mL. The GO in solution was electrodeposited on the GCE using a procedure similar to a previously reported protocol [41]. The GCEs were polished with 0.05 μm alumina slurry and sonicated in anhydrous ethanol and DI water prior to electrodeposition. The cyclic voltammetric (CV) reduction was performed in the GO solution under magnetic stirring, using a three-electrode system. The CV was run from a potential of 1 to −1.5 V at a scan rate of 50 mV/s for 18 cycles. Post-deposition, the reduced graphene oxide-GCE electrode (RGO-GCE) was washed with DI water and dried in nitrogen stream.

### 2.10. Attachment Chemistry for Cytosine Probes on the Electrode and Target Hybridization

The RGO-GCE was functionalized with amine terminated cytosine probes (CCCCCCCCCCCCCCC CCCCC/3’-NH_2_). The RGO-GCE was etched in 1 M NaOH at 1.5 V to activate the electrode surface and to create carboxylic acid functional groups on the electrodeposited graphene oxide (Figure 4) [42]. To convert the carboxyl groups on RGO-GCE to amine-reactive NHS esters for attachment to amine terminated probes [43], 10 μL of freshly prepared 100 mM Sulfo-NHS and 400 mM EDC in 0.1 M of MES buffer (pH = 5.9) was pipetted on the RGO-GCE electrode surface for 1 h and then washed with MES buffer [35,44]. Subsequently, 10 μL of 25 μM cytosine probes in 1 × PBS was pipetted on the activated RGO-GCE electrode surface for 1 h, followed by washing with 1 × PBS to wash off the excess unattached cytosine probes [35]. Finally, the hybridization reactions were performed by incubating the target (magnetic bead/*E. coli*/secondary bead complexes) solution on the probe-RGO-GCE electrode for 1 h. The electrode surface was subsequently washed with 1 × PBS before EC detection.

### 2.11. Fluorescent Microscopy Characterization of Probe-Target Hybridization

The magnetic bead/*E. coli*/secondary bead complexes hybridized on the RGO-GCE electrode was examined under a fluorescent microscope (4×, 500 ms exposure, Olympus IX81 inverted microscope, Olympus DP71 12-bit CCD color camera, FITC filter) using LCGreen (2 μL) intercalating dye (Idaho Technology Inc.). The extraction was also done from DI water with no *E. coli* O157:H7 as the starting sample and was used as the negative control. Another control involved fluorescent imaging of the electrode surface with magnetic bead/*E. coli*/secondary bead complexes without polyGs added to it (no target). Since polyGs specifically bind to the cytosine probes on the electrode surface, the absence of polyGs in the magnetic bead/*E. coli*/secondary bead complexes would enable evaluating any non-specific binding to the electrode surface. The images were analyzed using Olympus DP Controller imaging software (Melville, NY, USA).

### 2.12. EC Measurements

Initially, DPV measurements were run on the RGO-GCE electrodes with only cytosine probes attached, to record the baseline. Subsequently, the DPV detection was used to detect the target (magnetic bead/*E. coli*/secondary bead complexes) containing different concentrations of captured *E. coli* O157:H7 (0, 3, 20, 200, 300 CFUs) hybridized to the cytosine probes. Five consecutive DPV scans were performed to determine the guanine oxidation peak corresponding to each of the hybridized target. The differential value (S1–S5) was plotted for each target concentration (S1: first scan; S5: fifth scan). The DPV measurements (pulse size: 20 mV and scan rate: 5 mV/s) were conducted from 0.5 to 1.2 V (*vs*. Ag/AgCl) in 0.2 M acetate buffer solution (pH 5) containing 5 μM $\text{Ru}{\left(\text{bpy}\right)}_{3}^{2+}$ as the supporting electrolyte. During DPV, the effect of the charging current is minimized and hence enhanced signal-to-noise ratio can be achieved [45].

### 2.13. Pre-Concentration, IMS and EC Testing of E. coli O157:H7 in Waste Water Sample

To test the effectiveness of the *E. coli* O157:H7 detection process in simulated waste water, filtration, IMS and EC detection assay was run on waste water plant effluent (100 mL sample volume) from the local waste water treatment facility. Initially, vacuum filtration using a 30 μm nylon net filter (NY3004700, Millipore, Billerica, MA, USA) was employed to remove any solids >30 μm. Subsequently, the waste water was concentrated into 1 mL using vacuum filtration similar to the protocol for *E. coli* O157:H7 in PBS buffer samples. Next, IMS was performed to extract the *E. coli* O157:H7 from the 1 mL samples. Filtration, IMS extraction, and EC detection was performed to determine the amount of background *E. coli* O157:H7 in the waste water effluent samples. The waste water effluent was then seeded with 300 CFU *E. coli* O157:H7 and tested. Subsequently, 100 mL of the seeded waste water sample was autoclaved and the process was repeated to determine the signal generated by dead bacteria.

## 3. Results and Discussion

### 3.1. E. coli O157:H7 Extraction Efficiency Using Filtration and IMS

Three runs of *E. coli* O157:H7 extraction from 100 mL samples using vacuum filtration yielded an average percentage recovery of 47%. The filtration was employed before IMS to concentrate the *E. coli* O157:H7 from 100 mL samples into a 1 mL sample, because IMS on 100 mL samples directly resulted in only a 22% extraction efficiency. The low extraction percentage is likely due to the relatively low concentration of magnetic beads in the 100 mL sample volume. Increasing the number of beads to bring the concentration up to recommended levels would be cost prohibitive for the 100 mL samples. Pre-concentration using vacuum filtration is a cost-effective alternative for sample enrichment, which can also be incorporated into point-of-use systems [46]. The efficiency of capture of *E. coli* O157:H7 using IMS after vacuum filtration from different concentrations (500, 50, and 5 bacteria/mL in 1 mL 1× PBS sample volume) of *E. coli* O157:H7 was 95%, yielding an overall bacteria extraction efficiency of 46%.

### 3.2. Specificity of the E. coli O157:H7 IMS Process

Three runs of IMS using *E. coli* O157:H7 specific magnetic beads in non-specific pathogen samples (3000 CFUs *Salmonella*) yielded an average 0.4% extraction efficiency in comparison to the 95% for *E. coli* O157:H7 signifying that the IMS is highly specific to *E. coli* O157:H7.

### 3.3. Electrodeposition of Graphene Oxide on GCE

**Figure 5:**
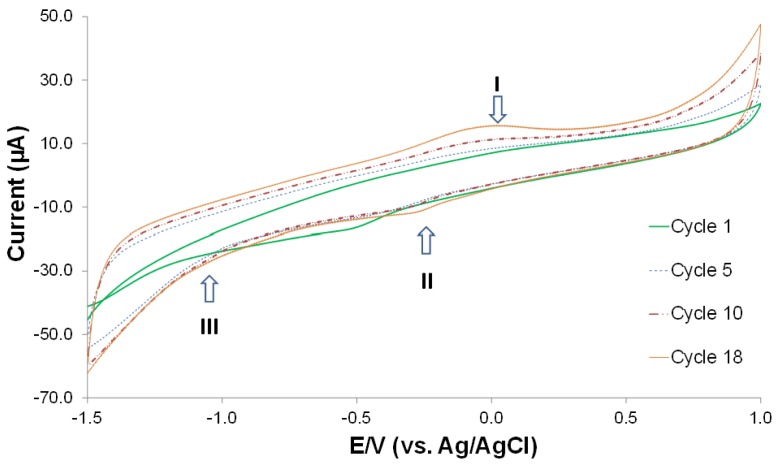
CV curve of graphene oxide electrodeposition on a GCE showing one anodic peak -I and two cathodic peaks -II and III.

Graphene oxide was deposited on the GCE electrodes in preparation for bacteria detection. Electrode modification by deposition of graphene oxide has been applied to a large number of EC biosensing applications [47–50]. The modification of the GCE by graphene oxide enhances the surface area, electron transfer kinetics, and enables attachment of probes by further surface modification of the graphene oxide layers [41,51]. Figure 5 shows the cyclic voltammetry of graphene oxide electrodeposition on a GCE, showing one anodic peak (I) and two cathodic peaks (II and III). The cathodic peak III is attributed to the electrochemical reduction of GO, and the anodic peak I and cathodic peak II are ascribed to the redox pair of some electrochemically active oxygen-containing groups on the graphene plane that are too stable to be reduced by the CV [41,52]. The increase in the peak currents with successive potential scans from cycle 1 to 18 is confirmation of the deposition of reduced graphene oxide on the bare GCE. The graphene electrodeposition happens on conducting surfaces only, and the resultant graphene coating is very stable due to its poor insolubility in common solvents [41].

### 3.4. Fluorescent Microscopy Confirmation of Probe-Target Hybridization

**Figure 6:**
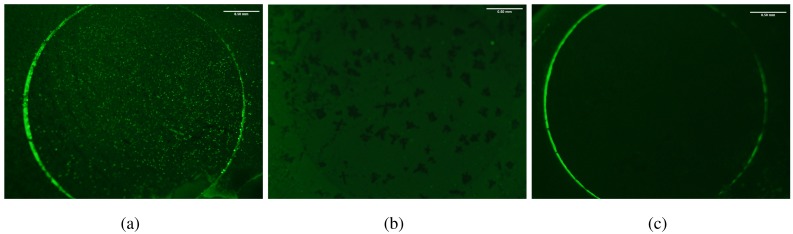
Fluorescent microscopy images of (a) Bound magnetic bead/*E. coli*/secondary bead complexes on RGO-GCE; (**b**) negative control 1 (DI water as starting sample- no *E. coli* present); and (**c**) negative control 2 (polyGs absent on the magnetic bead/*E. coli*/secondary bead complexes).

Cytosine probe attachment on the RGO-GCE was carried out followed by hybridization of the target magnetic bead/*E. coli*/secondary bead complexes. Fluorescence imaging was done to confirm the capture of magnetic bead/*E. coli*/secondary bead complexes on the cytosine probe functionalized RGO-GCE surface. The fluorescence images shown in Figure 6 generated using an LCGreen intercalating dye clearly show that the appropriate hybridization between the probe DNA and target polyG on the secondary beads has occurred. The number of bound beads was significantly higher than those for the negative control (essentially DI water with no *E. coli* O157:H7 as the starting sample) or the no target (polyGs absent on the magnetic bead/*E. coli*/secondary bead complexes) test. The results suggest that the general process is working and that the secondary beads bind as appropriate to the functionalized RGO-GCE surface and that minimal non-specific binding occurs.

### 3.5. In-Direct Electrochemical Detection of E. coli O157:H7

**Figure 7:**
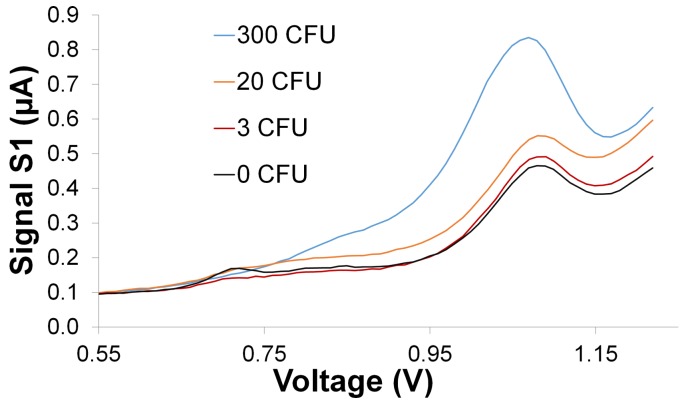
Absolute DPV signals (S1) corresponding to an order of magnitude change in concentration of *E.coli* O157:H7 from 3 to 300 CFUs. EC measurement condition: pulse size: 20 mV, scan rate: 5 mV/s, scan range 0.5 V to 1.2 V (vs. Ag/AgCl reference electrode). Supporting electrolyte: 0.2 M acetate buffer solution (pH 5) containing 5 μM $\text{Ru}{\left(\text{bpy}\right)}_{3}^{2+}$.

**Figure 8:**
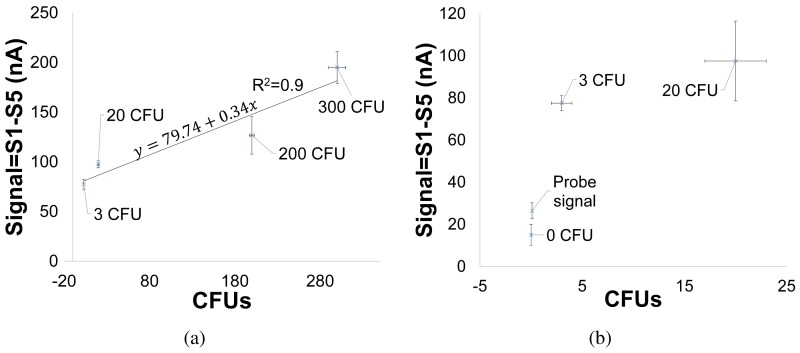
Relative DPV signals (S1-S5) corresponding to varying concentrations of *E. coli* O157:H7 in seeded 100 mL PBS buffer samples.

Electrochemical DPV was used to quantitatively measure the amount of hybridized polyG tags on the electrodes and hence indirectly measure the amount of captured *E. coli* O157:H7. The use of $\text{Ru}{\left(\text{bpy}\right)}_{3}^{2+}/\text{Ru}{\left(\text{bpy}\right)}_{3}^{3+}$ as an electron mediator during the oxidation of guanine (polyG) is well documented [53–57]. In the absence of any polyG, the background current/peak signal is due to the oxidation of $\text{Ru}{\left(\text{bpy}\right)}_{3}^{2+}$ at the electrode (RGO-GCE) surface. In the presence of polyG, the amplified peak signal during the first scan (S1) is due to the irreversible oxidation of guanine bases [55]. Hence the relative oxidation signals (S1-S5) increases as the concentration of polyG increases. Figure 7 shows the change in absolute DPV signals (S1) with an order of magnitude change in CFUs from 3 to 300 CFUs. These peak signals are observed between 1.06–1.07 V. In addition, a relatively smaller peak is seen at 0.7 V which is possibly due to some contaminants in the tested samples. Figure 8 shows the relative DPV signals (S1–S5) corresponding to varying concentrations of *E. coli* O157:H7 (0 to 300 CFUs enumerated by plate counting) in the initial seeded 100 mL PBS buffer samples.

The relative oxidation signal due to guanine increased from 0 to 300 CFUs. The standard deviation was found to be 56.5% for three successive 300 CFU measurements. From the Figure 8a, the calibration curve is linear in the range from 3–300 CFUs, with regression equation of y = 79.74 + 0.34*x* with *R*^2^ = 0.9. The detection limit was 3 CFU/100 mL with a signal-to-noise ratio of 3 (the noise being the probe only signal). The 0 CFU does give a signal of 15 nA which corresponds to the base signal due to $\text{Ru}{\left(\text{bpy}\right)}_{3}^{2+}$ in the electrolyte (Figure 8b). The average probe only signal (RGO-GCE with functionalized probes) was higher than the signal corresponding to 0 CFU (Figure 8b). This is because there is a drop in signal during DPV cycles due to passivation by acetate buffer in the electrolyte. This was confirmed by a drop in peak signals between first and second scans, seen during DPV performed with RGO-GCE electrodes in acetate buffer solution (not shown). Since the probe scans were initially run for all the electrodes before hybridized target scans was performed, there is a drop in signal for 0 CFU compared to probe only signal (Figure 8b).

### 3.6. Detection of E. coli O157:H7 in Simulated Waste Water

**Figure 9:**
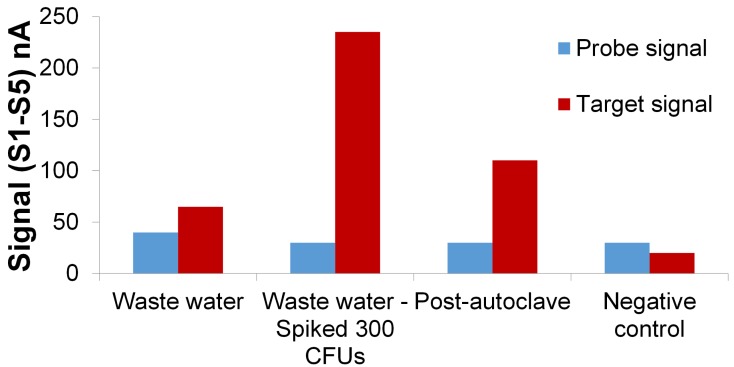
Electrochemical signal corresponding to *E. coli* O157:H7 in waste water effluent samples. Negative control is in the form of DI water without any *E. coli* O157:H7 in it. EC measurement condition: pulse size: 20 mV, scan rate: 5 mV/s, scan range 0.5 V to 1.2 V (*vs*. Ag/AgCl reference electrode). Supporting electrolyte: 0.2 M acetate buffer solution (pH 5) containing 5 μM $\text{Ru}{\left(\text{bpy}\right)}_{3}^{2+}$.

Our assay was able to detect *E. coli* O157:H7 in waste water plant effluent (Figure 9). The amount of native *E. coli* O157:H7 in waste water effluent samples was unknown. The initial test yielded a 65 nA signal. The waste water effluent was then seeded with 300 CFU *E. coli* O157:H7 and tested. The results in Figure 8, show the electrochemical signal at 225 nA post-seeding with 300 CFU *E. coli* O157:H7. The difference in signal corresponds to 225 − 65 = 180 nA which is 95% of signal corresponding to 300 CFUs tested in PBS buffer solution (Figure 8a). The negative control (DI water) gave a signal of 20 nA which corresponds to signal range for 0 CFUs in buffer. Post autoclaving the waste water sample gave a detection signal, indicating that dead bacteria were also detected. One possible solution to fix this would be to run an additional scan after a prescribed time (about 1 h) to gauge the amount of live bacteria.

## 4. Conclusions

The protocol utilizing the IMS of *E. coli* O157:H7 and subsequent electrochemical detection of polyG functionalized secondary beads was able to detect 3 CFU *E. coli* O157:H7 in 100 mL samples with a signal-to-noise ratio of 3. The detection time was approximately 2 h. A linear relationship was found between the *E. coli* O157:H7 concentration and the relative electrochemical signal in the 3–300 CFU range with *R*^2^ = 0.9. The IMS indicated a 95% extraction efficiency for *E. coli* O157:H7 with only a 0.4% non-specific capture. The overall extraction efficiency of *E. coli* O157:H7 from 100 mL samples was 46%. Detection of CFU levels below 3 CFU runs into statistical and repeatability issues especially in 100 mL samples. The detection limits of *E. coli* are two orders of magnitude better than what is reported in literature [4], when measured and demonstrated limits of detection are compared directly. The protocol was also able to detect *E. coli* O157:H7 in waste water samples. While not demonstrated in this work, the protocol can be easily modified for detecting multiple pathogens simultaneously by incorporating different oligonucleotide targets on the secondary beads and multiple electrodes (*i.e*., microarray) with corresponding complementary probes.
